# Mutational profiling of acute lymphoblastic leukemia with testicular relapse

**DOI:** 10.1186/s13045-017-0434-y

**Published:** 2017-03-02

**Authors:** Ling-Wen Ding, Qiao-Yang Sun, Anand Mayakonda, Kar-Tong Tan, Wenwen Chien, De-Chen Lin, Yan-Yi Jiang, Liang Xu, Manoj Garg, Zhen-Tang Lao, Michael Lill, Henry Yang, Allen Eng Juh Yeoh, H. Phillip Koeffler

**Affiliations:** 1 Cancer Science Institute of Singapore, National University of Singapore, Singapore, Singapore; 2 Division of Hematology/Oncology, Cedars-Sinai Medical Center, UCLA School of Medicine, Los Angeles, USA; 3 Department of Medical Oncology and Clinical Research, Cancer Institute (WIA), Adyar Chennai, India; 4 Department of Haematology, Singapore General Hospital, Singapore, Singapore; 5 Department of Pediatrics, Division of Hematology and Oncology, National University Health System, Singapore, Singapore

**Keywords:** Acute lymphoblastic leukemia, ALL, Testicular relapse, Extramedullary relapse

## Abstract

Relapsed acute lymphoblastic leukemia (ALL) is the leading cause of deaths of childhood cancer. Although relapse usually happens in the bone marrow, extramedullary relapse occasionally occurs including either the central nervous system or testis (<1–2%). We selected two pediatric ALL patients who experienced testicular relapse and interrogated their leukemic cells with exome sequencing. The sequencing results and clonality analyses suggest that relapse of patient D483 directly evolved from the leukemic clone at diagnosis which survived chemotherapy. In contrast, relapse leukemia cells (both bone marrow and testis) of patient D727 were likely derived from a common ancestral clone, and testicular relapse likely arose independently from the bone marrow relapsed leukemia. Our findings decipher the mutational spectra and shed light on the clonal evolution of two cases of pediatric ALL with testicular relapse. Presence of *CREBBP*/*NT5C2* mutations suggests that a personalized therapeutic approach should be applied to these two patients.

**Electronic supplementary material:**

The online version of this article (doi:10.1186/s13045-017-0434-y) contains supplementary material, which is available to authorized users.

To the editor

Relapsed acute lymphoblastic leukemia (ALL) is the leading cause of deaths of childhood cancer [1–3]. Although relapse usually occurs in the bone marrow (medullary), extramedullary relapse occasionally occurs, including either in the central nervous system or testis (<1–2%). Involvement of these organs is often associated with an inferior prognosis, perhaps because the blood-brain/blood-testis barrier hinders efficient delivery of chemotherapy, and/or the leukemic cells infiltrated in these immune-privileged sites may escape efficient immune surveillance. Currently, clonal origin and evolution of extramedullary relapse ALL remain poorly understood. To address this, we selected two pediatric ALL patients who experienced testicular relapse and interrogated their leukemic cells with exome sequencing (see Additional file 2: Supplementary Methods).

Patient D483 (5.6 years old at diagnosis) was treated as an intermediate-risk B-ALL [hyperdiploid:56,XY,+X,t(2;14)(p?13;q32),+4,+8,+9,+10,+14,+17,+18,+21,+21, absence of any well-known leukemic fusion oncogene]. He developed bone-marrow (96% blast) and testicular relapse 5 years after induction of remission. Mutations of *KRAS* (G12D) and *CREBBP* (S1436C, in histone-acetyltransferase HAT domain) were found in the founding leukemic clone at diagnosis and persisted in bone marrow and testis at relapse (Additional file 1: Table S1). *CREBBP* encodes a histone/non-histone acetyltransferases which is involved in regulation of glucocorticoid gene expression, its mutation contributes to prednisolone/dexamethasone (glucocorticoid) resistance [4, 5]. Mutation and copy-number deletion of *CREBBP* are frequent in B-cell lymphoma and ALL [3, 6] and are often associated with disease relapse. A missense mutation (R17Q) of *MEF2B* (MADS-box transcription enhancer factor-2) was found in both the bone marrow and testicular relapse samples. Missense mutation of *MEF2B* is frequently detected in diffuse large B-cell lymphoma and contributes to malignant transformation by regulating expression of the proto-oncogene BCL6 [7]. Other mutations included *EVX1* (homeobox protein) and *OTUD5* (regulates p53 stability by deubiquitinating p53) (Additional file 1: Table S1).

Second patient (case D727, 1.3 years old at diagnosis of B-ALL) harbored a MLL-AF9 fusion gene [t(9;11)] and was treated as a high-risk ALL (82.8% blast in peripheral blood). *MLL* fusion is often associated with infant-ALL and a poor prognosis. Complete remission was achieved after induction therapy; however, the patient relapsed (91% blast) after a 2.3-year remission. *NT5C2* gene (encodes a 5′-nucleotidase involved in purine metabolism) had two mutations in the relapse samples, differencing in their VAF in the bone marrow (34%) and testicle (5%) for R367Q mutation; while D407V mutation was present with a VAF of 7% in bone marrow and 36% in testicular relapse. These two *NT5C2* mutations occur as recurrent mutational hotspots in relapse-ALL and they have been functionally validated [8]. These mutations increase the NT5C2 inosine-5-monophosphate-nucleotidase activity; and therefore lead to resistance to one of the chemotherapeutic drugs, 6-mercaptopurine [8, 9] (part of child’s treatment). Additional mutations that occurred in this child’s ALL cells included *DUSP13* (phosphatase that regulates JNK/P38 phosphorylation), *MAPK8* (*JNK1*), *PPP1R3B* (protein phosphatase 1 regulatory subunit 3B), and *ALPK3* (alpha-kinase 3) (Additional file 1: Table S1).

To gain insight into the evolutionary trajectories of these two ALL cases, we analyzed mutational clustering of VAF and clonal evolution based on their sequencing data (Additional file 2: Supplementary Methods). Mutations shared at leukemic diagnosis and relapse represent early mutations and constitute the founding clone, while mutations occurring only at diagnosis in the marrow or only relapse samples of testicle/bone marrow likely were acquired later. For patient D483, the relapse leukemia directly evolved from the original leukemic clone at diagnosis; all mutations at diagnosis were persistent (Fig. 1a), and four additional missense-mutations [*MEF2B* (R17Q), *KCNG1* (L252V), *AIM1* (G109R), and *OTUD5* (G222D)] were acquired with different VAF at relapse in both bone marrow and testis, suggesting that both sites of relapse evolved from the same leukemic clone at diagnosis (Fig. 1b, c). In contrast, patient D727 had a proportion of mutations present at diagnosis which were absent at relapse, suggesting that the relapsed leukemia arose from an ancestral clone which existed before the overt leukemia at diagnosis. Analysis of mutational pattern and VAF suggests that relapse in patient’s testicle represents an independent subclone from the relapse in the bone marrow, albeit they share a common progenitor clone derived from the original ancestral clone (Fig. 2a–c). Of note, a fraction of mutations present at diagnosis persisted in the testicle but were absent in relapsed marrow, suggesting that the relapse ALL evolved following a parallel branching hierarchy instead of a linear acquisition path.

**Fig. 1:**
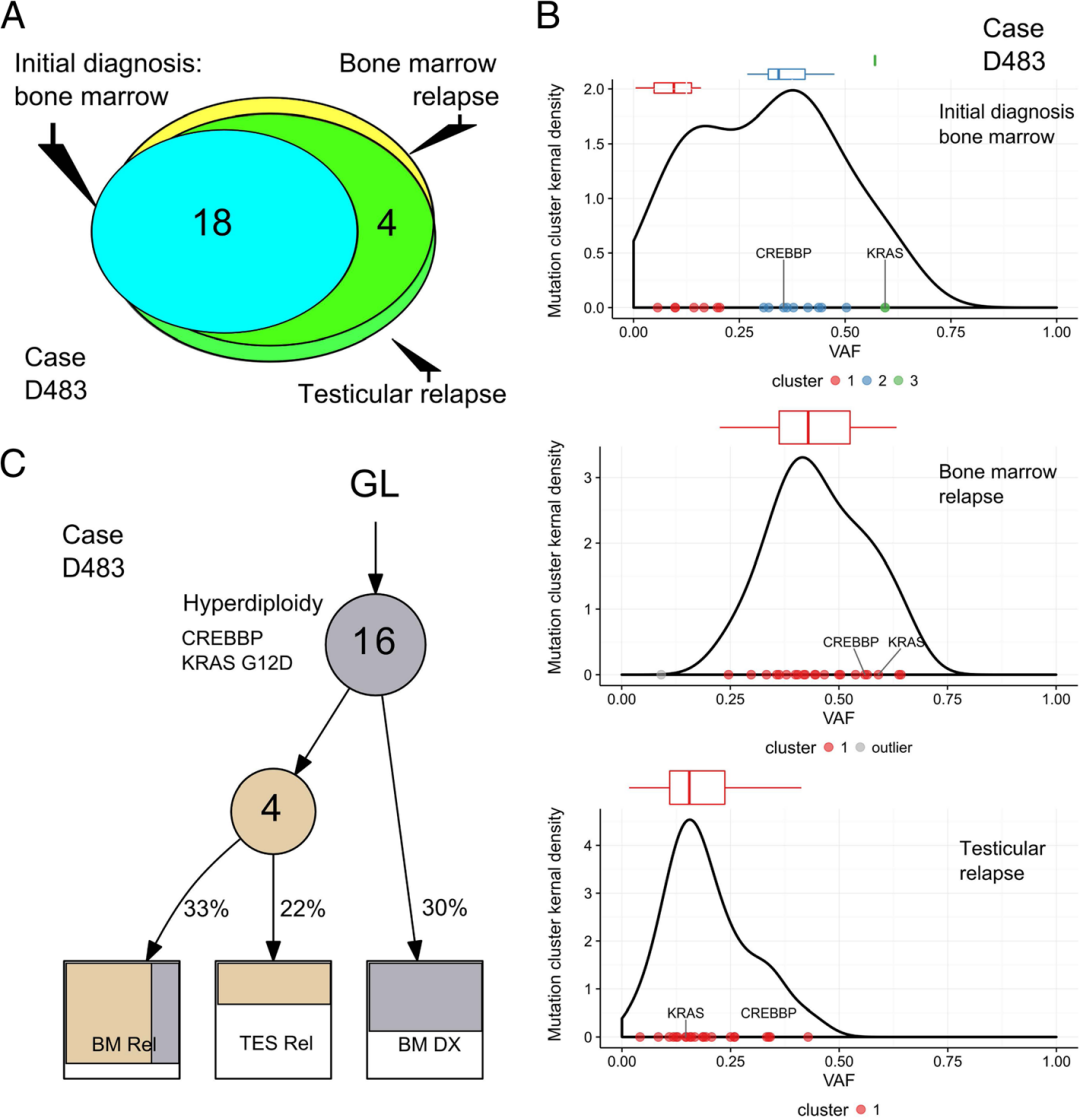
Clonal evolution of ALL in patient D483. **a** Venn diagram shows mutations that occurred at leukemic diagnosis, bone marrow relapse, and testicular relapse in patient. **b** Cluster of mutations at initial marrow diagnosis, relapse of bone marrow, and testicle. **c** Clonal evolution lineage tree and sample composition of case D483. Lineage tree was constructed based on the constraint network using LICHeE [10]. Each node (*circle*) represents a sub-population of leukemic cells. *Numbers inside circles* indicate number of shared single nucleotide variants (SNVs, including synonymous SNVs and filtered with outliers of mutation cluster), *numbers outside the circles* show the mean VAF of each cluster. *Color* in each sample indicates mutational groups in that sub-population of cells, and the subdivision in a sample suggest potential mixed lineage pattern. *GL* germline, *BM DX* bone marrow at diagnosis, *BM REL* bone marrow at relapse, *TES REL* relapse in the testis

**Fig. 2:**
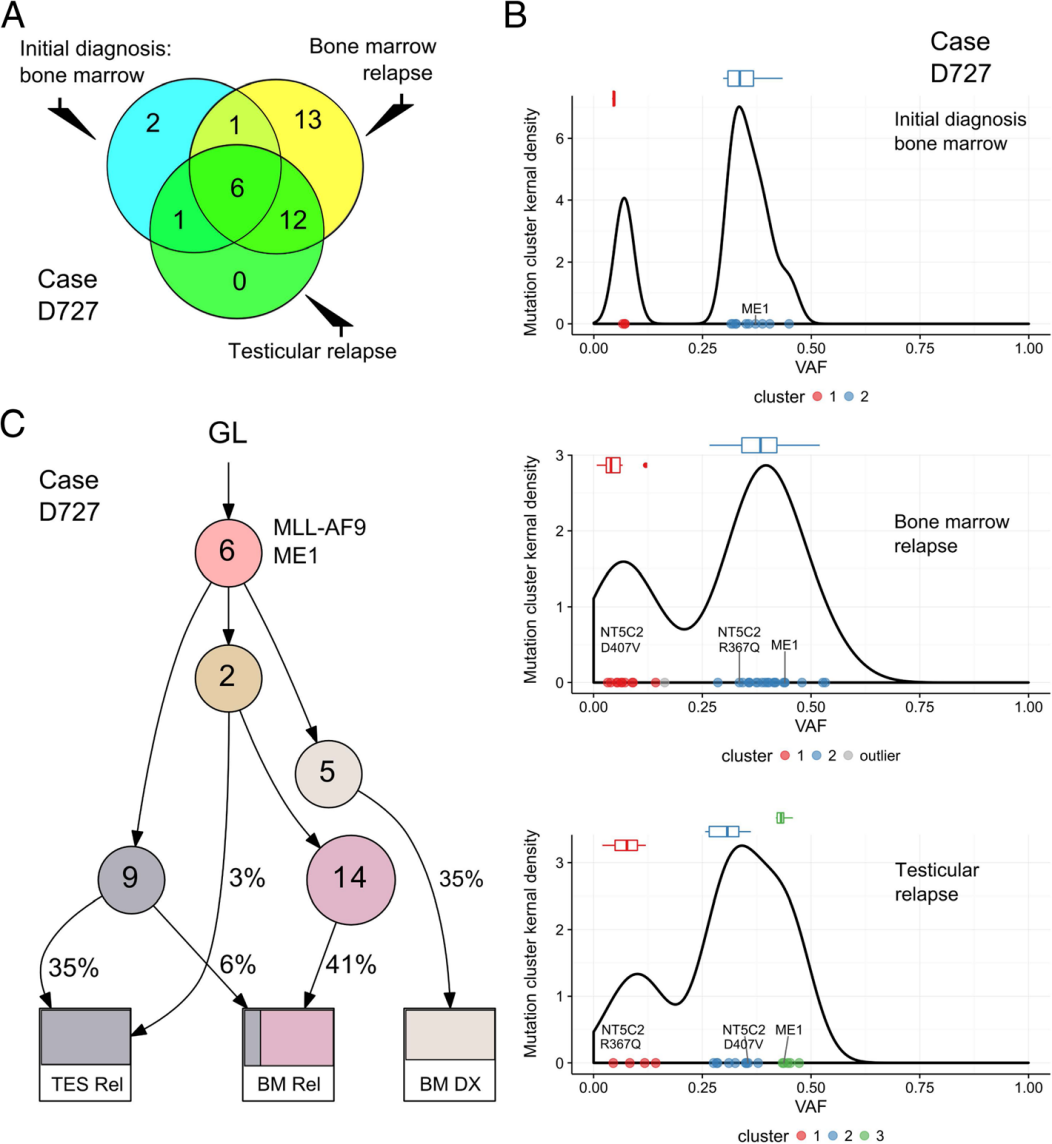
Clonal evolution of ALL in patient D727. **a** Venn diagram shows mutations that occurred at leukemic diagnosis and relapse of bone-marrow and testicle in patient D727. **b** Cluster of mutations at diagnosis (*DX*), bone marrow relapse (*REL*), and testicular relapse (*TES*). **c** Clonal evolution lineage tree and sample composition of case D727. Lineage tree was constructed based on constraint network using LICHeE [10]. Each node (*circle*) represents a sub-population of leukemic cells. *Numbers inside circles* indicate number of shared SNVs (including synonymous SNVs and filtered with outliers of mutation cluster), *numbers outside circles* show mean VAF of each cluster. *Color* in each sample indicates mutational groups in that sub-population of cells, and subdivision in a sample suggest a potential mixed lineage pattern. *GL* germline, *BM DX* bone marrow at diagnosis, *BM REL* bone marrow at relapse, *TES REL* relapse at testis

## Additional files

Additional file 1: Table S1. Variant allele frequency (VAF) of somatic mutations in both cases. (DOCX 24 kb)
Additional file 2: Supplementary Methods. (DOCX 14 kb)

## Abbreviations

ALL: Acute lymphoblastic leukemia
CR: Complete remission
DX: Diagnosis
REL: Relapse
VAF: Variant allele frequency
