# Supplementary material for: Mutational profiling of acute lymphoblastic leukemia with testicular relapse
Source: J Hematol Oncol. 2017 Mar 2;10:65. doi: 10.1186/s13045-017-0434-y (PMC5335697; doi:10.1186/s13045-017-0434-y)
Supplement: Additional file 1: — Table S1. Variant allele frequency (VAF) of somatic mutations in both cases. (DOCX 24 kb) [file 13045_2017_434_MOESM1_ESM.docx]

**Table S1 Variant allele frequency (VAF) of somatic mutations in both cases.**

VAF of somatic mutations of case D483

| Case D483 |  | VAF | | | |
| --- | --- | --- | --- | --- | --- |
| Gene | **Mutation** | **CR (%)** | **Initial DX BM (%)** | **REL BM (%)** | **TES REL (%)** |
| *UCMA* | p.R113Q | 0 | 10 | 42 | 16 |
| *C10orf12* | p.D1099Y | 0 | 10 | 36 | 8 |
| *KRAS* | p.G12D | 0 | 59 | 59 | 15 |
| *TPI1* | p.D4E | 0 | 44 | 47 | 13 |
| *ADAMTS17* | p.R222W | 0 | 38 | 42 | 19 |
| *CREBBP* | p.S1436C | 0 | 35 | 56 | 34 |
| *ATP2A3* | p.I298I | 0 | 20 | 30 | 17 |
| *MAPK4* | p.S461X | 0 | 20 | 38 | 19 |
| *ZNF407* | p.E1932K | 0 | 32 | 25 | 16 |
| *AMER3* | p.R463Q | 0 | 50 | 45 | 11 |
| *SON* | p.S830T | 0 | 17 | 54 | 33 |
| *SNED1* | p.R916K | 0 | 41 | 50 | 19 |
| *NKX3-2* | p.A113A | 0 | 14 | 36 | 43 |
| *POU4F2* | p.G77D | 0 | 44 | 57 | 4 |
| *TAP1* | p.V252A | 0 | 31 | 64 | 26 |
| *EVX1* | p.S117L | 0 | 6 | 40 | 13 |
| *STEAP4* | p.I433I | 0 | 36 | 44 | 12 |
| *FAM167A* | p.G146G | 0 | 59 | 64 | 34 |
| *MEF2B* | p.R17Q | 0 | 0 | 33 | 15 |
| *KCNG1* | p.L252V | 0 | 0 | 9 | 25 |
| *AIM1* | p.G109R | 0 | 0 | 50 | 26 |
| *OTUD5* | p.G222D | 0 | 0 | 41 | 21 |

CR: complete remission; DX BM: diagnosis bone marrow; REL BM: relapse bone marrow; TES REL: relapse in testis.

VAF of somatic mutations of case D727

| Case D727 |  | VAF | | | |
| --- | --- | --- | --- | --- | --- |
| Gene | **Mutation** | **CR (%)** | **Initial DX BM (%)** | **REL BM (%)** | **TES REL (%)** |
| *SLC5A8* | p.I186I | 0 | 44 | 38 | 38 |
| *ADORA2B* | p.T162M | 0.9 | 40 | 53 | 47 |
| *FZR1* | p.T112T | 0 | 39 | 0 | 0 |
| *ME1* | p.Y522C | 0 | 37 | 44 | 44 |
| *MUC17* | p.G2688G | 0 | 33 | 29 | 14 |
| *KIAA2022* | p.P642Q | 0 | 32 | 0 | 0 |
| *KRTAP1-3* | p.A141S | 0 | 32 | 40 | 0 |
| *PABPC1* | p.G35G | 0 | 7 | 9 | 28 |
| *MUC21* | p.T233S | 0 | 7 | 0 | 8 |
| *KRTAP1-1* | p.S48C | 0 | 7 | 14 | 12 |
| *NT5C2* | p.D407V | 0 | 0 | 7 | 36 |
| *NT5C2* | p.R367Q | 0 | 0 | 34 | 5 |
| *CEP164* | p.Q545Q | 0 | 0 | 3 | 35 |
| *DENND4A* | p.R1606Q | 0 | 0 | 6 | 35 |
| *ALPK3* | p.R239W | 0 | 0 | 7 | 31 |
| *ABCA12* | p.A1594A | 0 | 0 | 5 | 33 |
| *WDFY3* | p.A204A | 0 | 0 | 3 | 29 |
| *DCDC2* | p.S240L | 0 | 0 | 4 | 28 |
| *PPP1R3B* | p.T191M | 0 | 0 | 9 | 43 |
| *OR13F1* | p.I48M | 0 | 0 | 5 | 45 |
| *FREM1* | p.T1626A | 0.8 | 0 | 16 | 45 |
| *MAPK8* | p.P334P | 0 | 0 | 42 | 0 |
| *DUSP13* | p.R237G | 0 | 0 | 36 | 0 |
| *BBOX1* | p.P79P | 0 | 0 | 34 | 0 |
| *ANO6* | p.R659K | 0 | 0 | 44 | 0.4 |
| *GABPB1* | p.T197T | 0 | 0 | 53 | 0 |
| *BCO1* | p.S238N | 0 | 0 | 36 | 0 |
| *RYR1* | p.R1086H | 0 | 0 | 39 | 0 |
| *NLRP5* | p.V422M | 0 | 0 | 39 | 0 |
| *NPHP1* | p.R585Q | 0 | 0 | 41 | 0 |
| *COL5A2* | p.G498S | 0 | 0 | 37 | 1.4 |
| *CACNA1I* | p.R1449H | 0 | 0 | 40 | 0 |
| *SPATA16* | p.P330P | 0 | 0 | 42 | 0 |
| *MTNR1A* | p.D329N | 0 | 0 | 44 | 0 |
| *GSTA5* | p.S18F | 0 | 0 | 36 | 0 |
| *MSR1* | p.T80T | 0 | 0 | 48 | 0 |

CR: complete remission; DX BM: diagnosis bone marrow; REL BM: relapse bone marrow; TES REL: relapse in testis.
