# Supplementary material for: Mutational profiling of acute lymphoblastic leukemia with testicular relapse
Source: J Hematol Oncol. 2017 Mar 2;10:65. doi: 10.1186/s13045-017-0434-y (PMC5335697; doi:10.1186/s13045-017-0434-y)
Supplement: Additional file 2: — Supplementary Methods. (DOCX 14 kb) [file 13045_2017_434_MOESM2_ESM.docx]

**Supplementary Methods**

Currently, clonal origin and evolution of extramedullary relapse ALL remain poorly understood. Relapse ALL may derived from original leukemic clone at diagnosis, survive induction chemotherapy in an extramedullary site, grow and spread to bone marrow at clinical relapse. Alternatively, relapse leukemia may arise in the bone marrow, migrate to the extramedullary tissue and manifested as one of the sites of relapse, or both the bone marrow and testicular relapse without either one seeding the other at relapse.

To address this, we selected two pediatric ALL patients who experienced testicular ALL relapse, and interrogated their leukemic cells with next generation sequencing. Genomic DNA from bone marrow and testis were extracted using DNeasy Blood & Tissue Kit (Qiagen). Whole exome sequencing using Ion Proton™ System (100 X mean coverage) was performed to examine samples from both individuals (diagnosis, complete remission, relapse of bone-marrow relapse and testicule). Briefly, whole exome regions of each sample were amplified using Ion AmpliSeq™ HiFi Mix and Ion AmpliSeq™ Exome RDY plates. Resulting amplicons were treated with FuPa Reagent to remove primers and phosphorylate amplicons ends. After purification, amplicon libraries were ligated to Ion Xpress™ Barcode Adapters and sequenced using Ion Proton instrument. Generated reads were aligned to human genome build hg19 and somatic variants (SNV and indel) were identified using Torrent Variant Caller (Torrent Suite Software). For all unique variants, read counts were obtained using bam-readcount software (https://github.com/genome/bam-readcount). Analysis of clonal evolution and cancer lineages were performed using LICHeE (Lineage Inference for Cancer Heterogeneity and Evolution). Table containing VAFs was fed into LICHeE to infer tumor progression model. For heterogeneity estimation, Bayesian model-based clustering approach was used (mclust Version 5.2.1 for R).
